# Deletion of Neuropeptide Y Attenuates Cardiac Dysfunction and Apoptosis During Acute Myocardial Infarction

**DOI:** 10.3389/fphar.2019.01268

**Published:** 2019-10-24

**Authors:** Wei Huang, Qianhui Zhang, Hanping Qi, Pilong Shi, Chao Song, Yongsheng Liu, Hongli Sun

**Affiliations:** Department of Pharmacology, Harbin Medical University-Daqing, Daqing, China

**Keywords:** Neuropeptide Y, myocardial infarction, apoptosis, miR-499, FoxO4

## Abstract

Increasing neuropeptide Y (NPY) has been shown to be a risk factor for cardiovascular diseases. However, its role and mechanism in myocardial infarction (MI) have not yet been fully understood. H9c2 cells and neonatal rat ventricular myocytes with loss of function of NPY and rats with global knockout were used in this study. MI model of rats was induced by the ligation of left coronary artery, and the extent of MI was analyzed through echocardiographic, pathological, and molecular analyses. Our data demonstrated that NPY expression was significantly increased in MI rats and hypoxia/hydrogen peroxide (H_2_O_2_)-treated cardiomyocytes. At the same time, NPY-knockout rats exhibited a remarkable decrease in infarct size, serum lactate dehydrogenase activity, cardiomyocyte apoptosis, and caspase-3 expression and activity and a strong improvement in heart contractile function compared with MI rats. Meanwhile, NPY small interfering RNA (siRNA) inhibited the cell apoptosis in H_2_O_2_-treated H9c2 cells and hypoxia-treated neonatal rat ventricular myocytes. NPY deletion increased miR-499 expression and decreased FoxO4 expression in MI *in vivo* and *in vitro*. Moreover, NPY type 1 receptor antagonist BIBO3304 can reverse miR-499 decrease and FoxO4 increase in H_2_O_2_-induced cardiomyocytes. NPY siRNA inhibited cell apoptosis in H_2_O_2_-treated H9c2 cells that were reversed by miR-499 inhibitor. Additionally, FoxO4 was validated as the direct target of miR-499. Moreover, BIBO3304 and FoxO4 siRNA significantly increased the cell activity, inhibited the cell apoptosis, and decreased caspase-3 expression and activity in H_2_O_2_-treated cardiomyocytes that NPY presented the opposite effect. Collectively, deletion of NPY reduced myocardial ischemia, improved cardiac function, and inhibited cardiomyocyte apoptosis by NPY type 1 receptor–miR-499–FoxO4 axis, which provides a new treatment for MI.

## Introduction

Acute myocardial infarction (MI) is a myocardial perfusion obstruction disease, which is one of the leading causes of morbidity and mortality worldwide (Mozaffarian et al., 2016). Myocardial apoptosis is a type of cardiomyocyte death during MI, and additional progressive loss of surviving cardiomyocytes eventually leads to heart failure (Wencker et al., 2003; Chiong et al., 2011; Nabel and Braunwald, 2012). Therefore, it is very important to inhibit cardiomyocyte apoptosis in early MI to improve cardiac function, prevent ventricular remodeling, and reduce the incidence of cardiovascular events and is a key way to treat ischemic heart disease (Eltzschig and Eckle, 2011). However, the cellular mechanism of myocardial apoptosis induced by ischemia is complex and poorly understood.

Neuropeptide Y (NPY) is a peptide amine containing 36 residues that is widely distributed in the central system and peripheral nervous system and was first seen in the pig brain (Tatemoto, 1982; Pedrazzini et al., 2003). NPY plays an important role in regulating physiological functions such as emotions, cardiovascular and immune homeostasis, angiogenesis, cardiac remodeling, appetite, gastrointestinal motility, neuroendocrine axis, sympathetic nerve, and vagal nerve conduction (Grundemar and Hakanson, 1993; Wan and Lau, 1995; Shanks and Herring, 2013). NPY is the most abundant neuropeptide in the heart. It is found in the postganglionic sympathetic nerve cells, supplying the vascular system, endocardial and myocardial cells, as well as in cardiac ganglia and parasympathetic nerve cells (Gu et al., 1983; McDermott and Bell, 2007). However, in addition to playing an important role in normal physiological control mechanisms, it is also increasingly involved in the pathophysiological processes of cardiovascular diseases.

In humans and animals, stress conditions, such as exercise, hypoxia, cold exposure, tissue injury, ischemia, and hemorrhagic shock, will elevate levels of plasma NPY, which is associated with the pathophysiology of metabolic disorders (Pernow et al., 1987; Dvorakova et al., 2014). In addition, plasma NPY levels also increased in pathologic conditions of sympathetic neurosis, such as hypertension, left ventricular hypertrophy, MI, and heart failure (McDermott and Bell, 2007; Dvorakova et al., 2014; Tan et al., 2018). These effects increase the likelihood of NPY to treat cardiovascular disease. Animal studies have shown that NPY is released from the sympathetic nerve in heart during experimentally induced MI (Han et al., 1989). Clinical studies prior to percutaneous coronary angiography and modern medical treatment also showed an increase in “NPY-like activity” of peripheral venous during MI and left ventricular failure and was associated with 1-year mortality (Hulting et al., 1990; Ullman et al., 1994). A recent study found that patients who received initial percutaneous intervention had significantly higher NPY levels in their peripheral veins after MI and maintained high levels for at least 48 h (Cuculi et al., 2013). However, the role of NPY in the regulation of MI and its potential molecular mechanism remains to be studied.

In the current study, we used the MI model of rats and hypoxia/hydrogen peroxide (H_2_O_2_)-induced cardiomyocyte injury model to study whether NPY deletion has a protective effect on MI-induced cardiomyocyte apoptosis and myocardial dysfunction. Our findings suggest that NPY deletion may provide a new treatment for ischemic heart disease.

## Materials and Methods

### Ethics Statement

This study was carried out in accordance with the guidelines of the Institutional Animal Care and Use Committee of Harbin Medical University. The protocol was approved by the Animal Experimentation Ethics Committee of Harbin Medical University.

### Animal Experiments

NPY-knockout (NPY-KO) rats were graciously provided by Prof. Weidong Yong (Institute of Laboratory Animal Science, Chinese Academy of Medical Sciences & Peking Union Medical College, Beijing, China). Previous studies have shown how NPY-KO rats are produced (Qiu et al., 2016). In this study, male NPY-KO (220 ± 20 g) and their wild-type Wistar rats were used. As we previously described (Hang et al., 2015), the MI model was established by ligating the left anterior descending coronary artery. Rats were anaesthetized with ketamine–xylazine (100 mg/kg, 5 mg/kg, intraperitoneally). Sham-operated animals underwent the same procedure, but the coronary ligature was left untied. The electrocardiogram is recorded before and after ligation to confirm ischemia.

### Echocardiography

Echocardiography examination of rats was performed 3 days after MI. Echocardiograms are performed using an ultrasound machine Vevo2100 high-resolution imaging system (VisualSonics, Toronto, ON, Canada) and a 10-MHz imaging linear scanning probe transducer. Left ventricular ejection fraction (EF) and fractional shortening were calculated by M-mode recording method. Meanwhile, left ventricle (LV) end-diastolic diameter and LV end-systolic diameter dimensions were measured.

### H9c2 Cells and Neonatal Rat Ventricular Myocytes Culture and Transfection

The procedures to culture neonatal rat ventricular myocytes (NRVMs) were the same as described previously (Huang et al., 2016). NRVMs and H9c2 rat cardiomyocytes (American Type Culture Collection, Rockville, MD) were grown in Dulbecco’s modified Eagle’s medium (Hyclone, Logan, UT, USA) supplemented with 10% fetal bovine serum (Hyclone) under a humidified atmosphere of 95% air–5% carbon dioxide at 37°C, and subsequent experiments were performed 48 h after plating. The cells were subsequently treated with H_2_O_2_ (100 μM) for 4 h or hypoxia (1% O_2_) for 12 h to mimic MI.

MiR-499 mimic agent (50 nM) and the negative control microRNA (miRNA 50 nM) were synthesized by Guangzhou Ribo Biology Co., Ltd. The sequence of miR-499 mimic is 5’-UUAAGACUUGCAGUGAUGUUU-3’, and NC is 5’-UUUGUACUACACAAAAGUACUG-3’. NPY small interfering RNA (siRNA) (100 nM) and FoxO4 siRNA (100 nM) were purchased from Santa Cruz Biotechnology, USA. H9c2 cells or NRVMs (1 × 10^5^ per well) were starved in serum-free medium for 24 h prior to transfection with X-treme GENE siRNA transfection reagent (Roche, Germany) according to the manufacturer’s instructions. After 48 h transfection, H9c2 cells were treated with H_2_O_2_ for 4 h, and NRVMs were treated with 1% oxygen (O_2_), 5% carbon dioxide, and 94% dinitrogen for 12 h in a modular incubator separately. BIBO3304 (1 μM, Santa Cruz Biotechnology, USA) was preincubated for 12 h before H_2_O_2_ was treated.

### Measurement of Infarct Size

Three days after MI, triphenyltetrazolium chloride (Sigma-Aldrich) was stained to the heart, and the size of the infarction was measured. After washing out remaining blood, the heart was cut into 2-mm-thick slices below the ligature line and stained with 1% triphenyltetrazolium chloride at 37°C for 15 min. The infarct area is stainless, and the living area is red. The area of heart infarction was calculated using Image ProPlus 5.0 software (Media Cybernetics, Wokingham, UK).

### Quantitative Real-Time Reverse Transcription-Polymerase Chain Reaction

According to the manufacturer’s agreement, TRIZOL reagent (Invitrogen, USA) was used to extract the total RNA from H9c2 cells or heart tissues after different treatments. The levels of NPY and caspase-3 (glyceraldehyde 3-phosphate dehydrogenase as the internal control), miR-1, miR133, miR-208a, and miR-499 mRNA (U6 as the internal control) were determined using SYBR Green to integrate into Roche LightCycler^®^480 Real Time PCR system (Roche, USA). The sequences of primers were: miR-1 forward: 5’-UGGAAUGUAAAGAAGUGUGUAU-3’ and reverse: 5’-AUACACACUUCUUUACAUUCCA-3’; miR-133 forward: 5’- UUGGUCCCCUUCAACCAGCUGU-3’ and reverse: 5’- AGCUGGUUGAAGGGGACCAAAU-3’; miR-208a forward: 5’- GTCATCTAGAAAGCTTGATGCAGGAAAGAGCTTTGG’ and reverse: 5’- TGACAGATCTCAGCTGA CATCCTCTAGGCTGGGGTT-3’; miR-499 forward:5’- GGTCCAGACTGGGGTCCCAGC-3’ and reverse: 5’- GCATGCCGCAGTGGTTAGGGA-3’; U6 forward: 5’-GCTTCGGCACATATACTAAAAT-3’ and reverse: 5’-CGCTTCACGAATTTGCGTGTCAT-3’; glyceraldehyde 3-phosphate dehydrogenase forward: 5’-AAGAAGGTGGTGAAGCAGGC-3’ and reverse: 5’-TCCACCACCCAGTTGCTGTA-3’; NPY forward: 5’- GGCCAGATACTACTCCGCTCTGCG-3’ and reverse: 5’- TTCACAGGATGAGATGAGATGTG-3’; caspase-3 forward: 5’- CTCGCTCTGGTACGGATGTG-3’ and reverse: 5’- TCCCATAAATGACCCCTTCATCA-3’; FoxO4 forward: 5’- CTTTCTGAAGACTGGCAGGAATGTG-3’ and reverse: 5’- GATCTAGGTCTATGATCGCGGCAG-3. Target mRNA (2^-ΔΔCT^) quantity was obtained by normalizing to endogenous reference and relative to a calibrator (average of the control or sham samples).

### Luciferase Assay

To generate reporter vectors bearing miRNA-binding sites, the 3-untranslated region (3′-UTR) of FoxO4 and its mutation type were synthesized by Sangon (Shanghai, China). The construct was inserted into multiple cloning sites downstream of the luciferase gene (SacI and HindIII sites) in the pMIR-REPORT luciferase miRNA expression reporter vector (Ambion, USA). For the luciferase assay, 0.1 μg of luciferase reporters containing 3′-UTR were cotransfected with miR-499-5p mimic or miR-499-5p inhibitor or NC into HEK-293 cells using lipofectamine 2000 (Invitrogen, USA). As an internal control, 10 ng of renilla luciferase reporters was also included. Forty-eight hours after transfection, the cells were collected, and dual luciferase activities were measured by a luminometer according to the manufacturer’s instructions.

### Immunocytochemistry

Immunochemical analysis was performed to detect NPY expression in heart tissues. In short, the hearts were fixed with 4% paraformaldehyde (pH 7.4) for 48 h. Heart tissues slices (5 mm thick) were incubated with 3% hydrogen peroxide in methanol to remove the endogenous peroxidase activity and then treated with normal serum to block nonspecific binding. Subsequently, the slices were incubated with anti-NPY primary antibody (rabbit, 1:200) at 4°C overnight and secondary antibody for 1 h. The sections were visualized with a diaminobenzidine and counterstained with hematoxylin.

### Cell Viability Assay

The cells were treated as designated in 96 plates and were subsequently incubated with 20-µl 3-(4,5-dimethylthiazolyl-2)-2,5-diphenyltetrazolium bromide (0.5 mg/ml) for 4 h. The medium was carefully removed and was rocked for 10 min after 200-µl dimethyl sulfoxide was added to each well. The absorbance values were detected at 490 nm using an Infinite M200 microplate spectrophotometer (Tecan, Salzburg, Austria).

### Detection of Neuropeptide Y Level

According to the manufacturer’s instructions, NPY levels were detected using ELISA kits (Nanjing Jiancheng Bioengineering Institute, Nanjing, China).

### Terminal Deoxynucleotidyl Transferase Deoxyuridine Triphosphate Nick End Labeling Staining

According to the manufacturer’s instructions, the apoptosis of H9c2 cells, NRVMs, and the LV was detected using terminal deoxynucleotidyl transferase deoxyuridine triphosphate nick end labeling (TUNEL) fluorescence FITC kit (Roche, USA) by the TUNEL staining method. After TUNEL staining, 4’,6-diamidino-2-phenylindole (1:100, Beyotime Biotechnology, China) was used to stain nuclei solution. Fluorescence staining was observed with a Confocal Laser Scanning Microscope (FV1000, Olympus, Japan). The apoptotic rate was calculated as TUNEL-positive cells per field.

### Caspase-3 and Lactate Dehydrogenase Activity Assay

Caspase-3 activity kit (Beyotime Institute of Biotechnology, Jiangsu, China) and serum lactate dehydrogenase (LDH) activity kit (Nanjing Jiancheng Bioengineering Institute, Nanjing, China) were used to determine caspase-3 and LDH activity, as described in the previous study (Huang et al., 2016).

### Western Blot

Total protein was extracted from the H9c2 cells as described in previous study (Huang et al., 2016). Briefly, proteins were separated by electrophoresis on sodium dodecyl sulfate polyacrylamide gels and transferred moist to nitrocellulose filter membranes. Membranes were incubated with anti-FoxO4 (1:100, mouse monoclonal; Santa Cruz, USA) antibody overnight at 4°C with the following secondary antibodies (LI-COR Biosciences, Lincoln, NE, USA) for 1 h at room temperature in the dark the next day. The images were captured by the Odyssey CLx Infrared Imaging System (LI-COR Biosciences). Anti-β-actin (1:1,000, mouse polyclonal; Santa Cruz, CA, USA) antibody was an internal control.

### Data Analysis

The data are represented by means ± SEM. The statistical analyses were used by one-way analysis of variance (for groups of ≥3) and Student’s test (for two groups). In all cases, *P < 0.05* was considered to be statistically significant. The data were analyzed using GraphPad Prism 5.0.

## Results

### Neuropeptide Y Level Was Elevated in Myocardial Infarction

**Figure 1:**
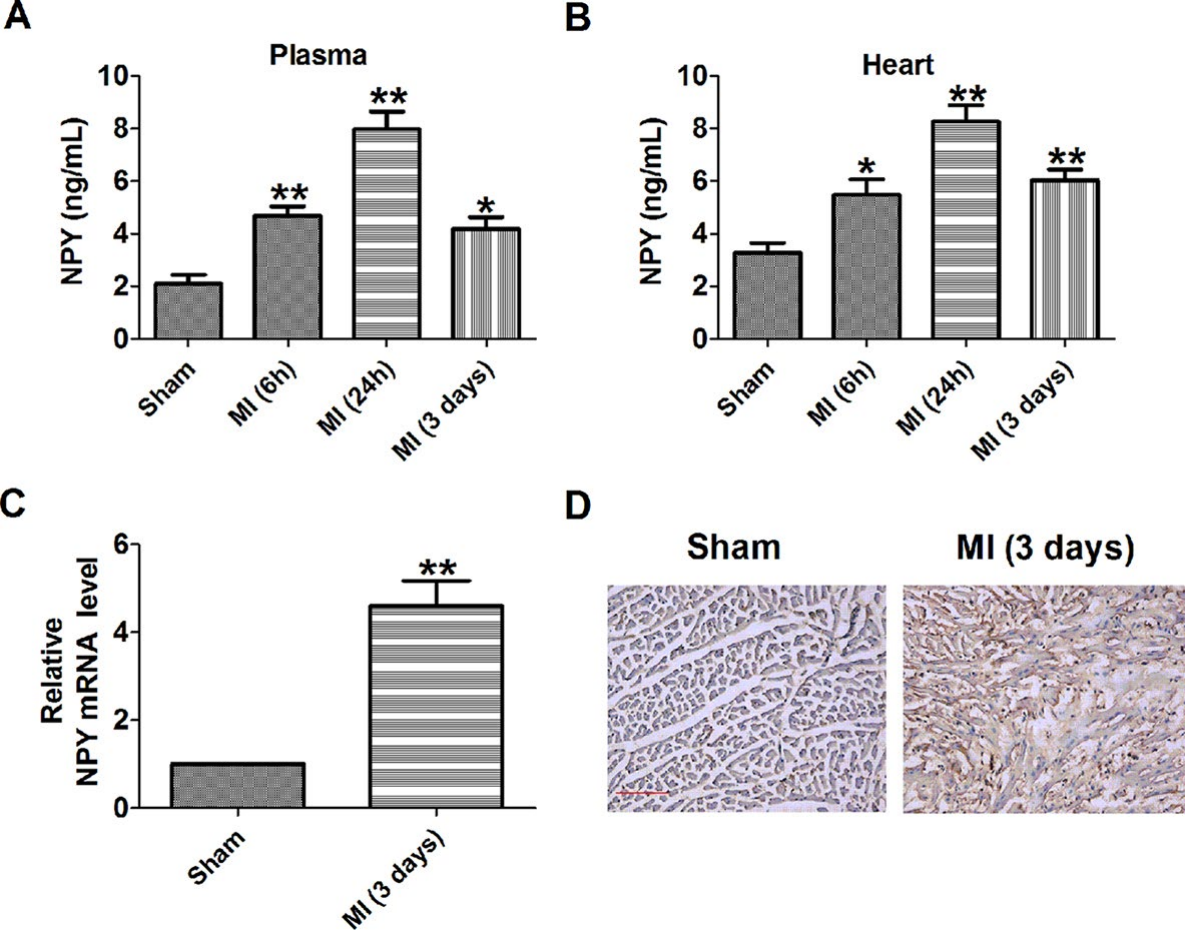
Level of neuropeptide Y (NPY) is increased in the plasma and heart of rat with myocardial **infarction**. **(A**, **B)** NPY level in the plasma and heart by immunoassay (n = 6). **(C)** The relative mRNA level of NPY (n = 6). **(D)** Immunocytochemistry analysis of NPY was examined in myocardial infarction myocardium (×100). Scale bar = 50 μm. Brown stain represented positive signal (n = 4). *P < 0.05, **P < 0.01 vs sham.

First, we examined the plasma and heart NPY level in MI rats. The ELISA analysis showed significant increases in plasma and heart NPY level of 6 and 24 h and 3 days after MI, with the highest level being 24 h (**Figures 1A, B**). At the same time, we observed a significant increase in heart NPY mRNA level of 3 days after MI compared with sham rats (**Figure 1C**). Immunochemistry analysis showed a similar result that the expression of NPY was increased in heart of MI rats (**Figure 1D**). Thus, these results suggested that NPY level was elevated in MI.

### Neuropeptide Y Knockout Decreases Infarct Size and Improves Cardiac Function of Infarcted Heart in Rats

**Figure 2:**
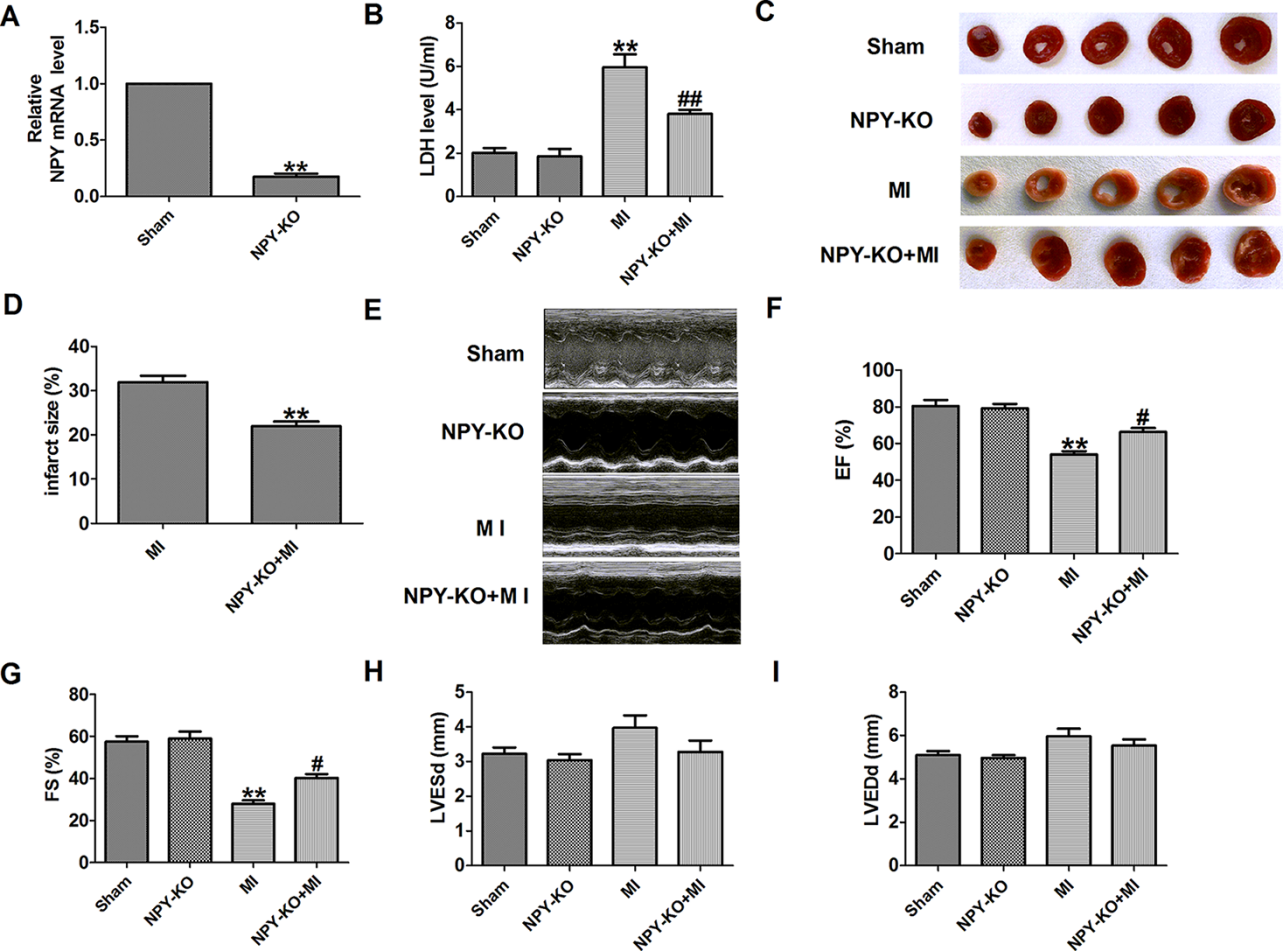
Effect of neuropeptide Y (NPY) knockout on lactate dehydrogenase (LDH) activity, cardiac infarct size, and cardiac function in rats 3 days post- myocardial infarction. **(A)** The relative mRNA level of NPY (n = 6). **(B)** Serum LDH activity (n = 4). **(C)** Representative images showing infarct areas in cross-section slices. **(D)** Statistical analysis of IA/LV ratio (n = 6). IA: infarct area, LV: left ventricles. **(E)** Representative photographs of heart function. **(F)** Ejection fractions (EF) (n = 6). **(G)** Fractional shortening (FS) (n = 6). **(H)** LV end-diastolic diameter (LVEDd) (n = 6). **(I)** LV end-systolic diameter (LVESd) (n = 6). **P < 0.01 versus sham; ^#^ *P* < 0.05, ^##^ *P* < 0.01 versus myocardial infarction.

First, NPY expression was detected in heart tissues of NPY-KO rats. NPY mRNA level was significantly decreased in NPY-KO rats compared with the sham group (**Figure 2A**). LDH is an important marker of MI, so serum LDH activity was detected. We found that serum LDH activity was significantly increased at 3 days post-MI that was decreased in NPY-KO rats compared with the sham group (**Figure 2**). We later found that NPY-KO significantly reduced the infarct size in MI (**Figures 2C, D**). Therefore, NPY-KO displayed a protective effect against ischemic injury. In addition, echocardiography showed a significant decrease in EF and fractional shortening of MI hearts, indicating impaired heart function (**Figures 2E–G**). NPY-KO attenuated the deterioration of left ventricular function in MI (**Figures 2E–G**). However, LV end-diastolic diameter and LV end-systolic diameter were unchanged by NPY-KO in MI (**Figures 2H, I**). It is worth noting that there were no significant abnormalities in serum LDH activity, infarct size, and cardiac function in NPY-KO rats compared with the sham groups (**Figures 2B–I**). These data indicated that NPY-KO has a protective effect on ischemic injury and can significantly relieve cardiac dysfunction during MI.

### Neuropeptide Y Knockout Inhibited Apoptosis in Ischemic Myocardium

**Figure 3:**
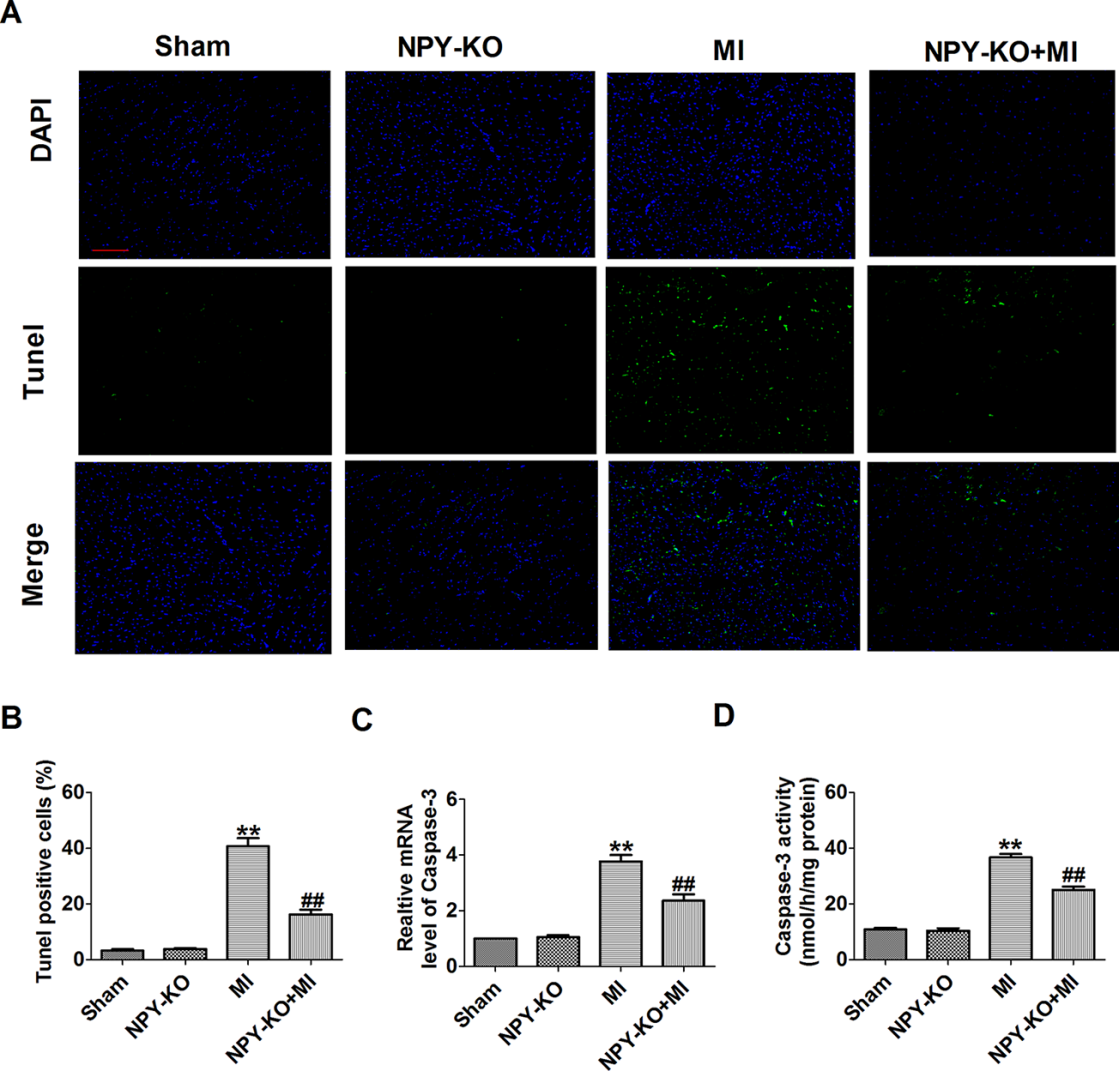
Effect of neuropeptide Y (NPY) knockout on cardiomyocyte apoptosis in rats 3 days post-myocardial infarction. **(A)** Effects of NPY knockout on cardiac apoptosis were evaluated by terminal deoxynucleotidyl transferase deoxyuridine triphosphate nick end labeling staining (nucleus stained in blue with 4’,6-diamidino-2-phenylindole and apoptotic cells stained in green). **(B)** The percentage of terminal deoxynucleotidyl transferase deoxyuridine triphosphate nick end labeling-positive cell in different groups (n = 4, 100×). Scale bar = 100 μm. **(C)** The mRNA level of caspase-3 (n = 6). **(D)** Caspase-3 activity (n = 6). **P < 0.01 versus control, ^##^ *P* < 0.01 versus myocardial infarction.

We further examined the apoptosis of cardiomyocyte by TUNEL staining. TUNEL-positive cells were increased in myocardium of MI rats compared with sham rats (**Figures 3A, B**). NPY-KO significantly inhibited cardiomyocyte apoptosis in MI (**Figures 3A, B**). Caspase-3 is a known key downstream protease that performs the apoptotic cascade (Porter and Janicke, 1999). We found an increase in caspase-3 mRNA level and activity of MI rats (**Figures 3C, D**). As expected, this elevation of caspase-3 mRNA level and activity in MI were blocked by NPY-KO (**Figures 3C, D**). Notably, NPY-KO rats did not exhibit marked abnormalities in cardiomyocyte apoptosis and caspase-3 mRNA level and activity compared with the sham groups (**Figures 3A–D**). The results showed that NPY-KO inhibited apoptosis of myocardial ischemic injury. NPY-KO inhibited apoptosis of ischemia injury.

### Neuropeptide Y Small Interfering RNA Prevented Hydrogen Peroxide-Induced Cardiomyocyte Apoptosis

**Figure 4:**
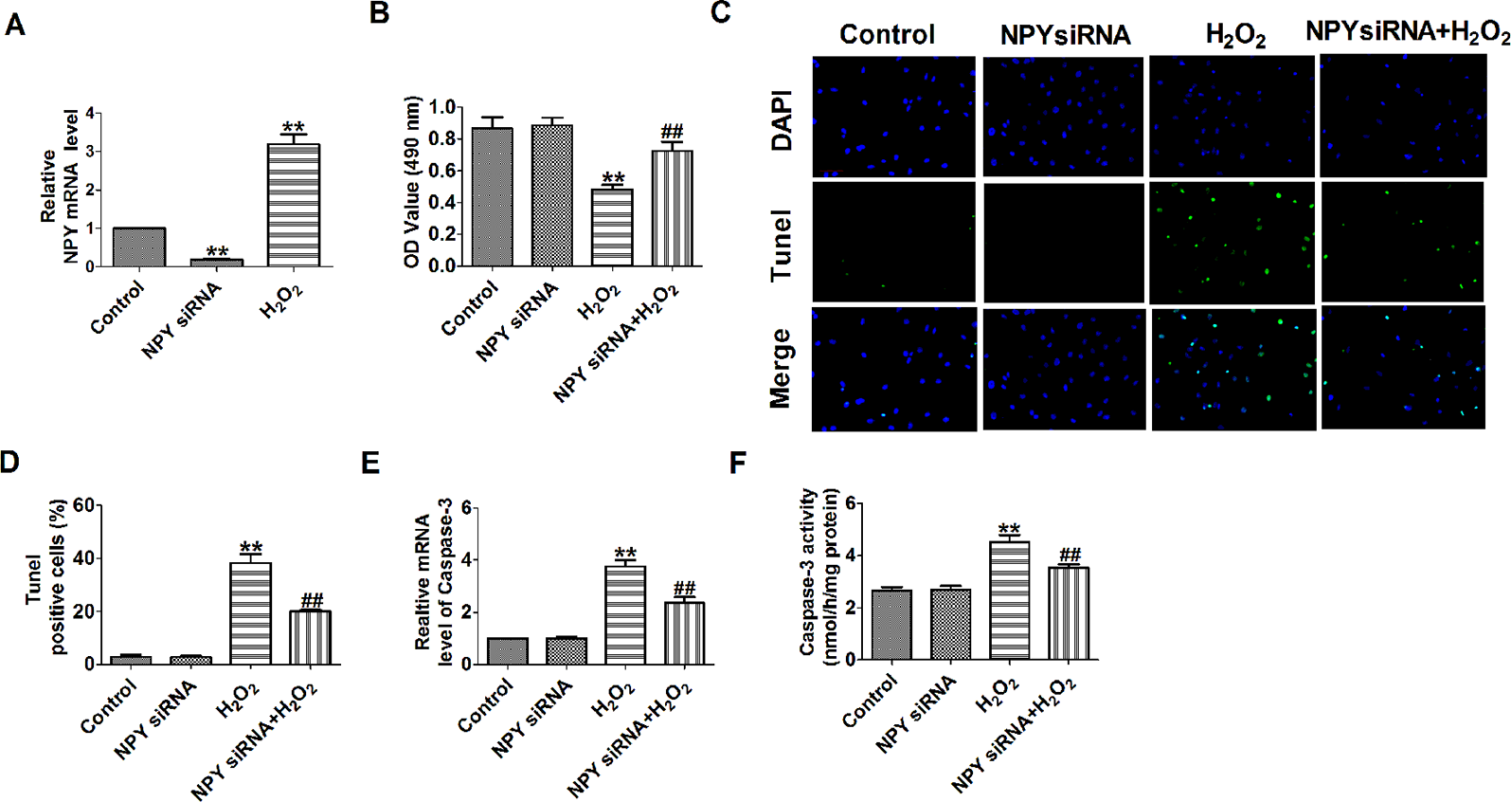
Effect of neuropeptide Y (NPY) small interfering RNA on cardiomyocyte apoptosis in response to hydrogen peroxidase. **(A)** The relative mRNA level of NPY (n = 6). **(B)** MTT assay (n = 6). **(C)** Representative images of terminal deoxynucleotidyl transferase deoxyuridine triphosphate nick end labeling staining of cardiomyocyte showing the apoptotic cells. **(D)** Statistical results of terminal deoxynucleotidyl transferase deoxyuridine triphosphate nick end labeling-positive cells per field (n = 4, 100×). Scale bar = 100 μm. **(E)** The mRNA level of caspase-3 (n = 6). **(F)** Caspase-3 activity (n = 6). **P < 0.01 versus control; ^##^ *P* < 0.01 versus hydrogen peroxidase.

Based on the earlier discussed results, we then aimed to assess the effects of NPY deletion on cell apoptosis. NPY mRNA level was significantly decreased in NPY siRNA group and was increased after cells were subjected to 100-μM H_2_O_2_ for 4 h (**Figure 4A**). H_2_O_2_ reduced cardiomyocyte viability, increased the number of TUNEL-positive cells and caspase-3 mRNA level and activity (**Figures 4B–F**). Compared with H_2_O_2_, NPY siRNA significantly increased the cell viability and decreased the number of TUNEL-positive cells and caspase-3 mRNA level and activity in H_2_O_2_-induced cardiomyocytes (**Figures 4B–F**). These results showed that NPY siRNA prevented cell apoptosis in H_2_O_2_-induced cardiomyocytes.

### Neuropeptide Y Small Interfering Rna Prevented Hypoxia-Induced Cardiomyocyte Apoptosis

**Figure 5:**
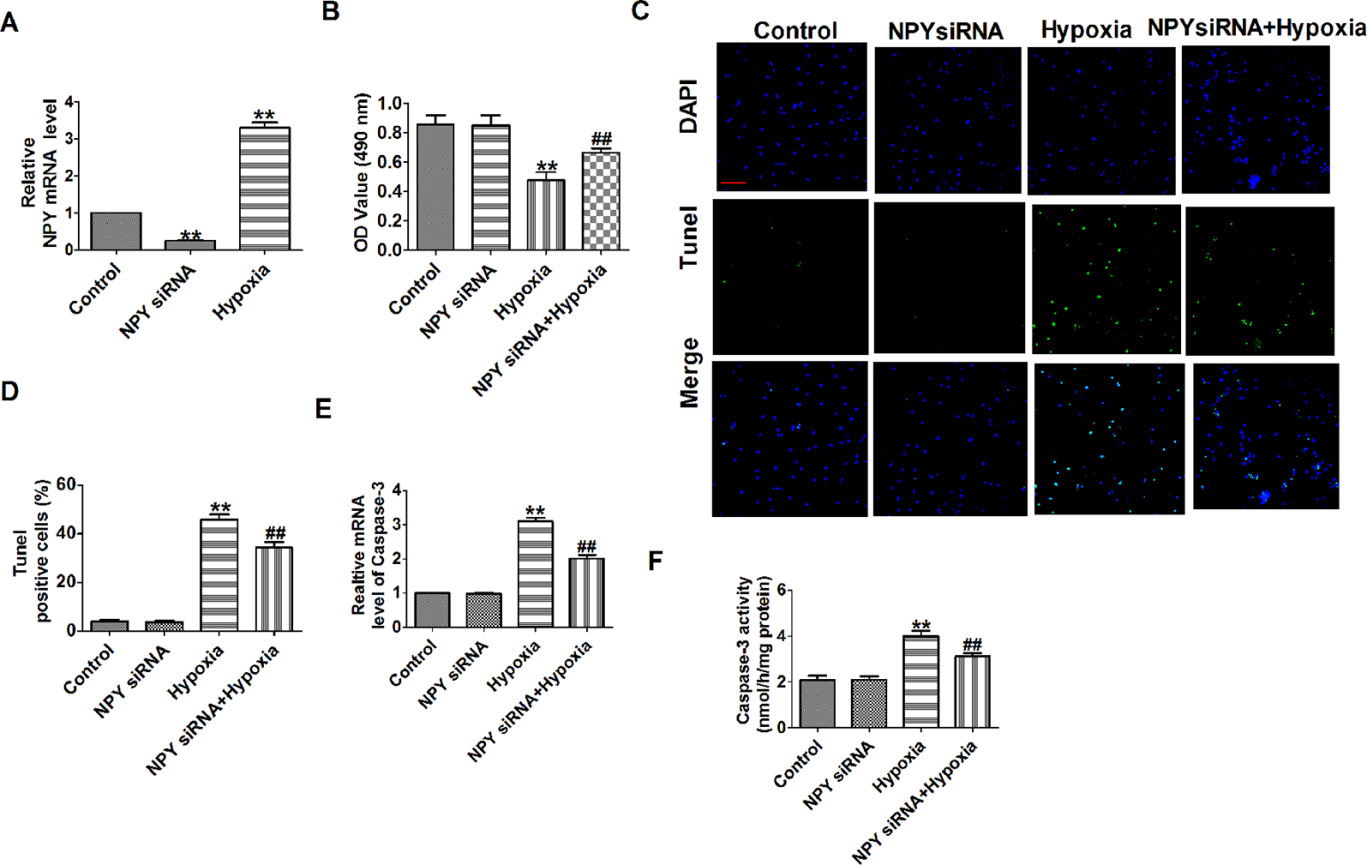
Effect of neuropeptide Y (NPY) small interfering RNA on cardiomyocyte apoptosis in response to hypoxia. **(A)** The relative mRNA level of NPY (n = 6). **(B)** MTT assay (n = 6). **(C)** Representative images of terminal deoxynucleotidyl transferase deoxyuridine triphosphate nick end labeling staining of cardiomyocyte showing the apoptotic cells. **(D)** Statistical results of terminal deoxynucleotidyl transferase deoxyuridine triphosphate nick end labeling-positive cells per field (n = 4, 100×). Scale bar = 100 μm. **(E)** The mRNA level of caspase-3 (n = 6). **(F)** Caspase-3 activity (n = 6). **P < 0.01 versus control; ^##^ *P* < 0.01 versus hypoxia.

NPY mRNA level was significantly decreased in NPY siRNA group and was increased after cells subjected to hypoxia (1% O_2_) for 12 h (**Figure 5A**). Hypoxia reduced cardiomyocyte viability, increased the number of TUNEL-positive cells and caspase-3 mRNA level and activity, which were restored by NPY siRNA in hypoxia-induced cardiomyocytes (**Figures 5B–F**). These results showed that NPY siRNA prevented cell apoptosis in hypoxia-induced cardiomyocytes.

### Neuropeptide Y-NPY1R Regulates Mir-499 and Foxo4 in Myocardial Infarction Model

**Figure 6:**
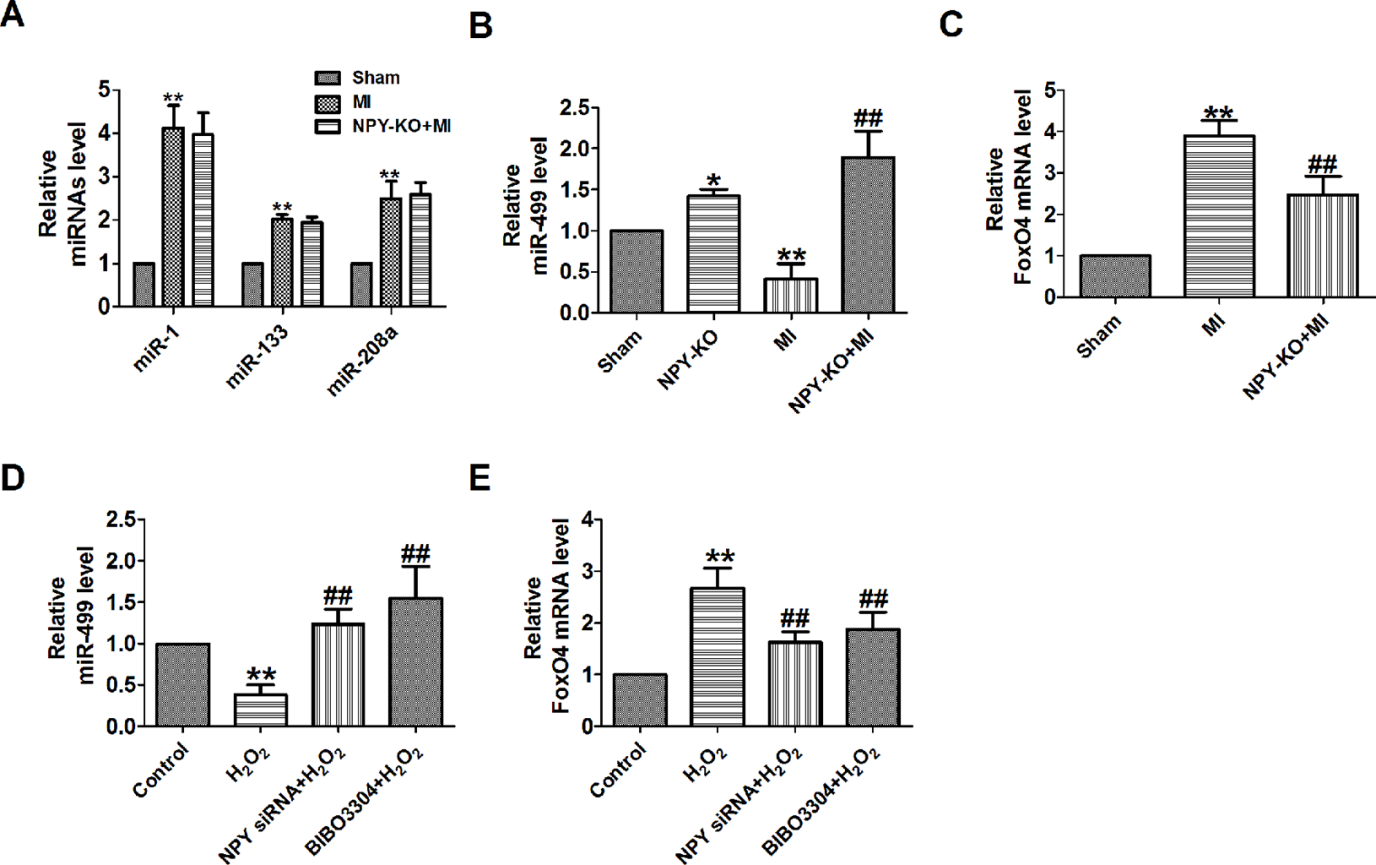
MiR-499 and FoxO4 is mediated by neuropeptide Y (NPY) and NPY1R in myocardial infarction (MI). **(A)** The relative level of the miRNAs in MI. **(B)** The relative level of the miR-499 in MI. **(C)** The relative mRNA level of FoxO4 in MI. **(D)** The relative level of the miR-499 in cardiomyocytes. **(E)** The relative mRNA level of FoxO4 in cardiomyocytes. n = 4. A-C: *P < 0.05, **P < 0.01 versus sham, ^##^ *P* < 0.01 versus MI; D&E: **P < 0.01 versus control; ^#^ *P* < 0.05, ^##^ *P* < 0.01 versus hydrogen peroxidase

A study has confirmed that NPY can regulate miR-30a in an *in vitro* model of Alzheimer’s disease (Croce et al., 2013). FoxO4 is a member of the ubiquitously expressed fork head (Fox) transcription factor O family that also includes FoxO1, O3, and O6. FoxO proteins regulate a variety of biological processes including oxidative stress response, metabolism, immunity, and apoptosis (Eijkelenboom and Burgering, 2013). In order to explore whether NPY mediates MI by miRNAs and FoxO4, we will focus on some of the miRNAs that have been predicted to be reliable markers of early MI in human and are cardiac specific or enriched in heart (Chistiakov et al., 2016). We found that miR-499 was decreased and FoxO4 was increased in MI *in vivo* and *in vitro*, which were reversed by NPY deletion (**Figures 6A–E**).

NPY causes coronary microvascular constriction and reduced EF following ST-elevation MI *via* NPY1R that is a predominant receptor subtype in the heart (Chottova Dvorakova et al., 2008; Herring et al., 2019). So, to see whether NPY1R was involved in NPY/miR-499 and NPY/FoxO4 axis, we determined the mRNA level of miR-499 by NPY1R antagonist in MI *in vitro*. We found that miR-499 and FoxO4 were also regulated by NPY1R antagonist BIBO3304 in MI *in vitro* as NPY siRNA (**Figures 6D, E**).

**Figure 7:**
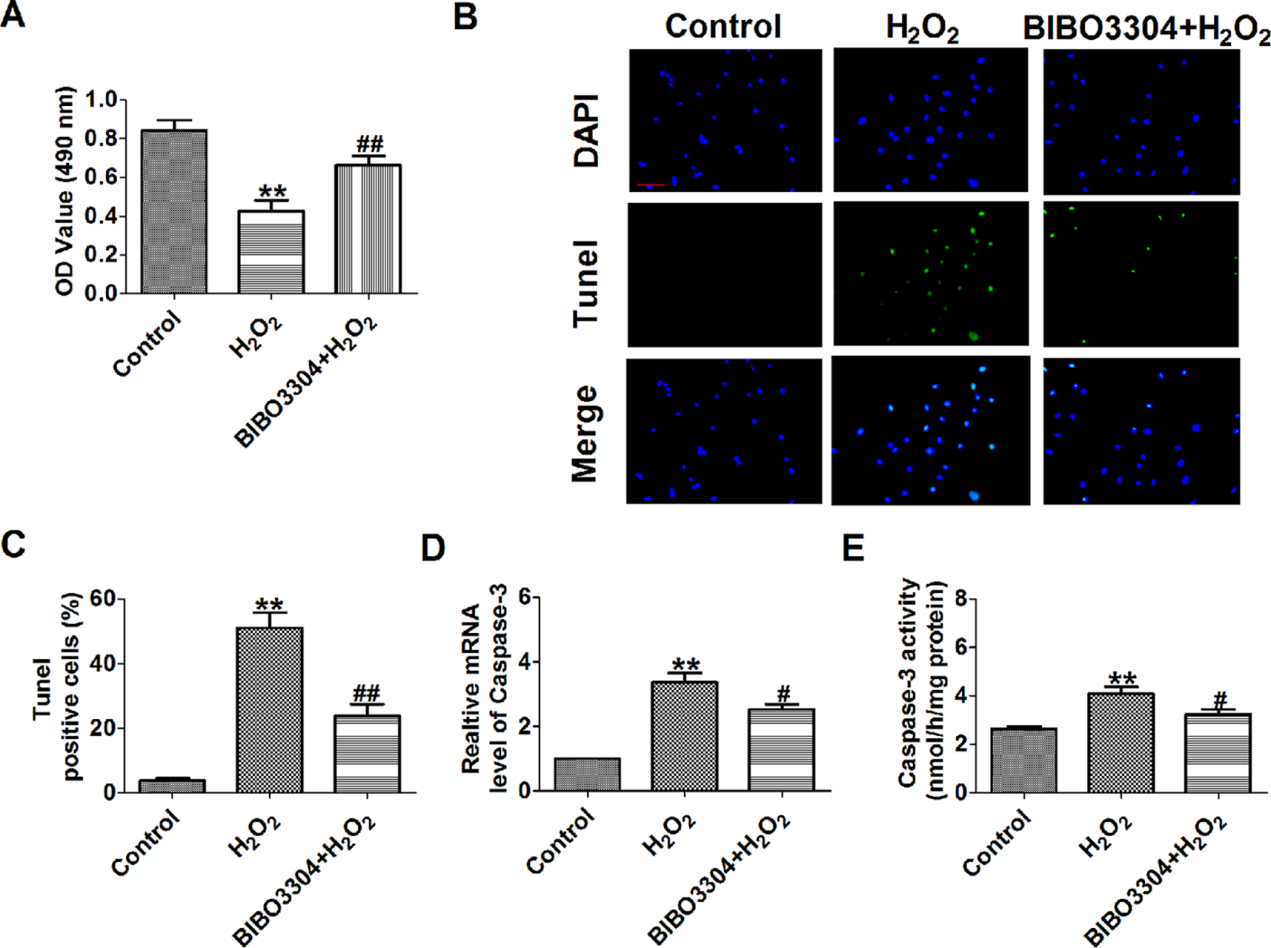
Effect of NPY1R antagonist BIBO3304 on cardiomyocyte apoptosis in response to hydrogen peroxidase. **(A)** MTT assay (n = 6). **(B)**. Representative images of terminal deoxynucleotidyl transferase deoxyuridine triphosphate nick end labeling staining of cardiomyocyte showing the apoptotic cells. **(C)**. Statistical results of terminal deoxynucleotidyl transferase deoxyuridine triphosphate nick end labeling-positive cells per field (n = 4, 100×). Scale bar = 100 μm. **(D)** The mRNA level of caspase-3 (n = 6). **(E)** Caspase-3 activity (n = 6). **P < 0.01 versus control; ^#^ *P* < 0.05, ^##^ *P* < 0.01 versus hydrogen peroxidase.

NPY significantly increases infarct area in relation to the area at risk during myocardial ischemia–reperfusion (I/R) injury, which is blunted by NPY1R antagonist BIBO3304 (Herring et al., 2019). So, we want to see whether NPY1R antagonist BIBO3304 inhibits H_2_O_2_-induced cardiomyocyte apoptosis. We found that BIBO3304 had a similar beneficial effect in H_2_O_2_-induced cardiomyocytes as NPY siRNA including a significantly recoverd cell viability and decreased the number of TUNEL-positive cells and caspase-3 mRNA level and activity (**Figures 7A–E**).

**Figure 8:**
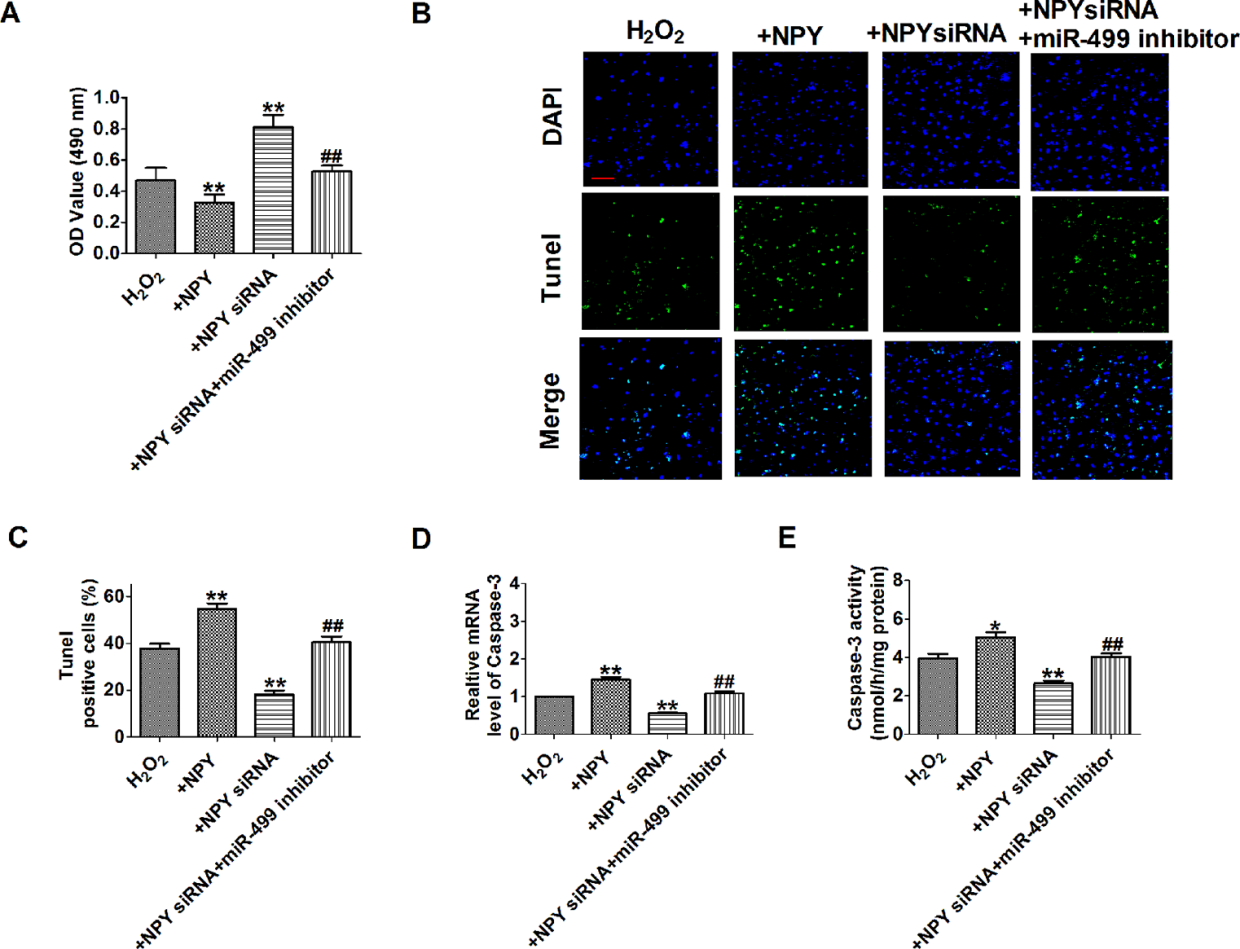
Neuropeptide Y (NPY) small interfering RNA up-regulate miR-499 expression and protected cardiomyocytes against hydrogen peroxidase (H_2_O_2_)-induced apoptosis. **(A)** MTT assay (n = 6). **(B)**. Representative images of terminal deoxynucleotidyl transferase deoxyuridine triphosphate nick end labeling staining of cardiomyocyte showing the apoptotic cells. **(C)**. Statistical results of terminal deoxynucleotidyl transferase deoxyuridine triphosphate nick end labeling-positive cells per field (n = 4, 100×). Scale bar = 100 μm. **(D)** The mRNA level of caspase-3 (n = 6). **(E)** Caspase-3 activity (n = 6). *P < 0.05, **P < 0.01 versus H_2_O_2_; ^##^ *P* < 0.01 versus NPY siRNA + H_2_O_2_.

We further examined signaling mechanism in which NPY siRNA mediated downregulation of miR-499. The results showed that NPY siRNA remarkably inhibited cell apoptosis and NPY promoted cell apoptosis in H_2_O_2_-induced cardiomyocytes (**Figures 8A–E**). NPY siRNA inhibited cell apoptosis that were reversed by cotransfection of miR-499 inhibitor in H_2_O_2_-induced cardiomyocytes (**Figures 8A–E**). These data implied that NPY siRNA mediated miR-499 on H_2_O_2_-induced cardiomyocyte apoptosis.

### Interplay Between Mir-499 and Foxo4

**Figure 9:**
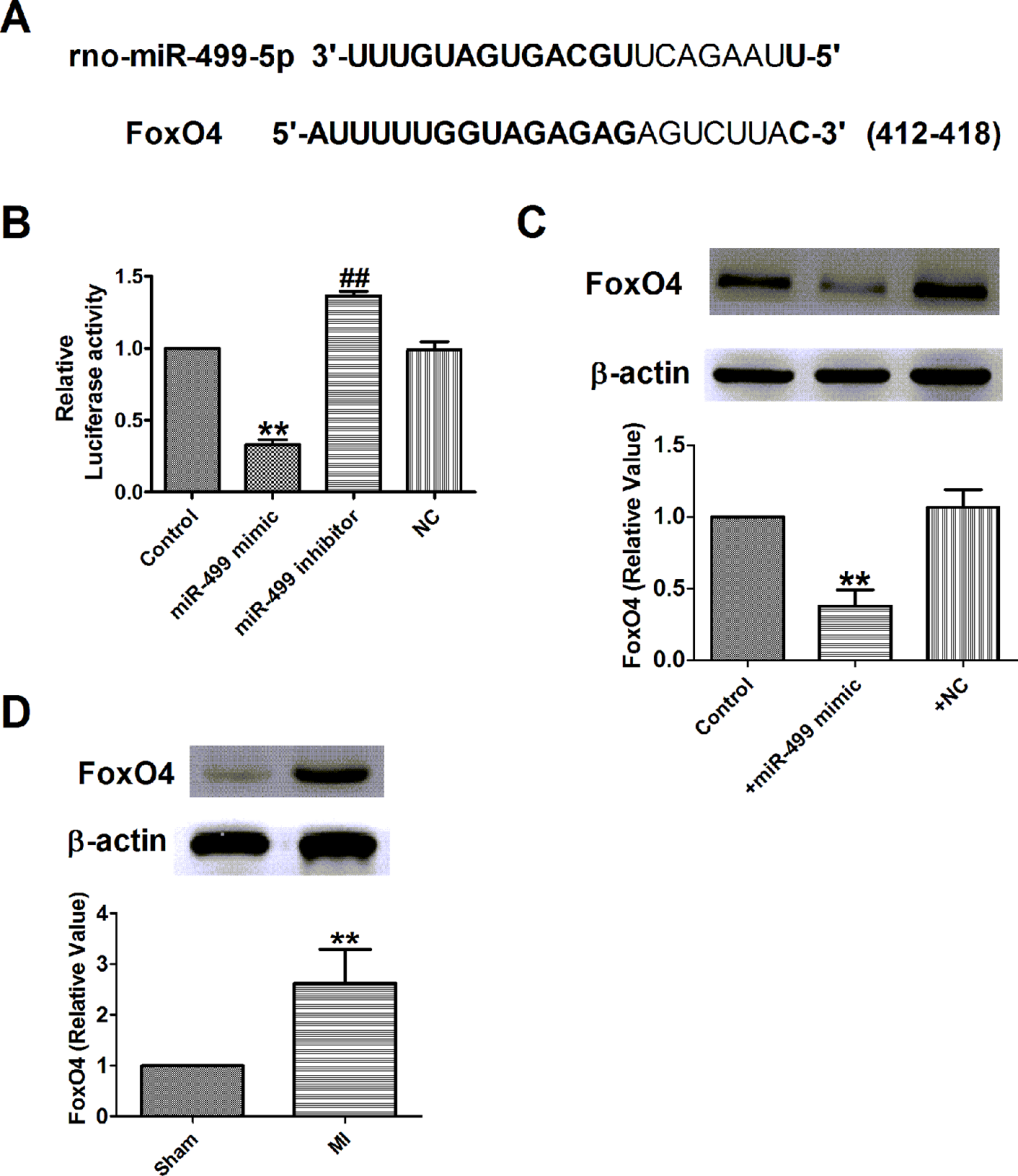
FoxO4 was the direct target of miR-499 in myocardial infarction *in vitro*. **(A)** Sequence alignment show between miR-499 and the binding sites in the 3’UTR of the FoxO4 gene. **(B)** The interaction between miR-499 and its binding sites in the 3’UTR of FoxO4 was examined by luciferase assay in HEK293 cells. **(C)** The expressions of FoxO4 protein were measured *in vitro*. **(D)** The expressions of FoxO4 protein were measured in myocardial infarction. B&C: **P < 0.01 versus control, ^##^ *P* < 0.01 versus miR-499 mimic; D: **P < 0.01 versus sham. n = 4.

A study showed that miR-499 promoted cellular invasion and tumor metastasis in colorectal cancer by targeting FoxO4 (Liu et al., 2011) and inhibited H_2_O_2_-induced cardiomyocyte apoptosis (Wang et al., 2014). We further examined the possible interaction between miR-499 and FoxO4 in H9c2 cells. As illustrated in **Figure 9A**, the 3’-UTR of FoxO4 has one binding site of miR-499. Luciferase assay was used to identify the inhibitory effect of miR-499 on FoxO4 (**Figure 9B**). The results demonstrated that miR-499 dramatically inhibited the luciferase activity of the constructed plasmid containing 3´-UTR of FoxO4 (**Figure 9B**). Next, we detected the protein expression of FoxO4 after being transfected with miR-499 mimic or NC. In our present study, we found that protein expression of FoxO4 was significantly inhibited by miR-499 mimic (**Figure 9C**) and validated the relationship between miR-499 and FoxO4. Last, we found that the protein expression of FoxO4 was increased in rat MI models (**Figure 9D**).

**Figure 10:**
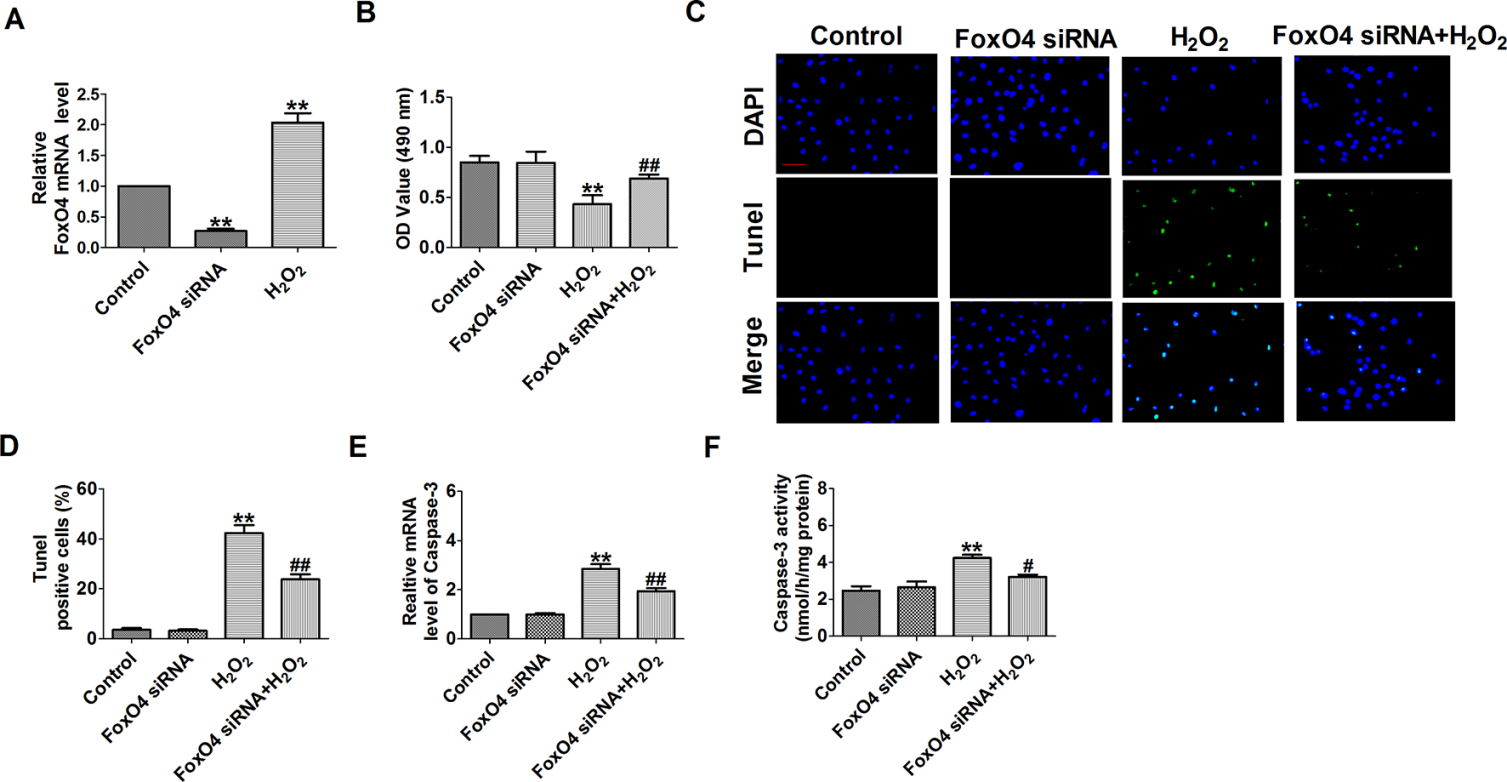
Effect of FoxO4 small interfering RNA on cardiomyocyte apoptosis in response to hydrogen peroxide. **(A)** The relative mRNA level of FoxO4 (n = 6). **(B)** MTT assay (n = 6). **(C)** Representative images of terminal deoxynucleotidyl transferase deoxyuridine triphosphate nick end labeling staining of cardiomyocyte showing the apoptotic cells. **(D)** Statistical results of terminal deoxynucleotidyl transferase deoxyuridine triphosphate nick end labeling-positive cells per field (n = 4, 100×). Scale bar = 100 μm. **(E)** The mRNA level of caspase-3 (n = 6). **(F)** Caspase-3 activity (n = 6). **P < 0.01 versus control; ^#^ *P* < 0.05, ^##^ *P* < 0.01 versus hydrogen peroxide.

FoxO4 promotes early inflammatory response upon MI *via* endothelial Arg1 and myocardial I/R injury and I/R-induced myocardial apoptosis (Zhu et al., 2015; Yu et al., 2018). So, we need to see whether FoxO4 inhibits H_2_O_2_-induced cardiomyocyte apoptosis. First, FoxO4 mRNA level was significantly decreased in FoxO4 siRNA group and was increased in H_2_O_2_-induced cardiomyocytes (**Figure 10A**). Next, FoxO4 siRNA significantly increased the cell viability and decreased the number of TUNEL-positive cells and caspase-3 mRNA level and activity in H_2_O_2_-induced cardiomyocytes (**Figures 10B–F**). Taken together, these findings suggested that miR-499 that inhibited FoxO4 expression prevented cardiomyocyte apoptosis.

## Discussion

**Figure 11:**
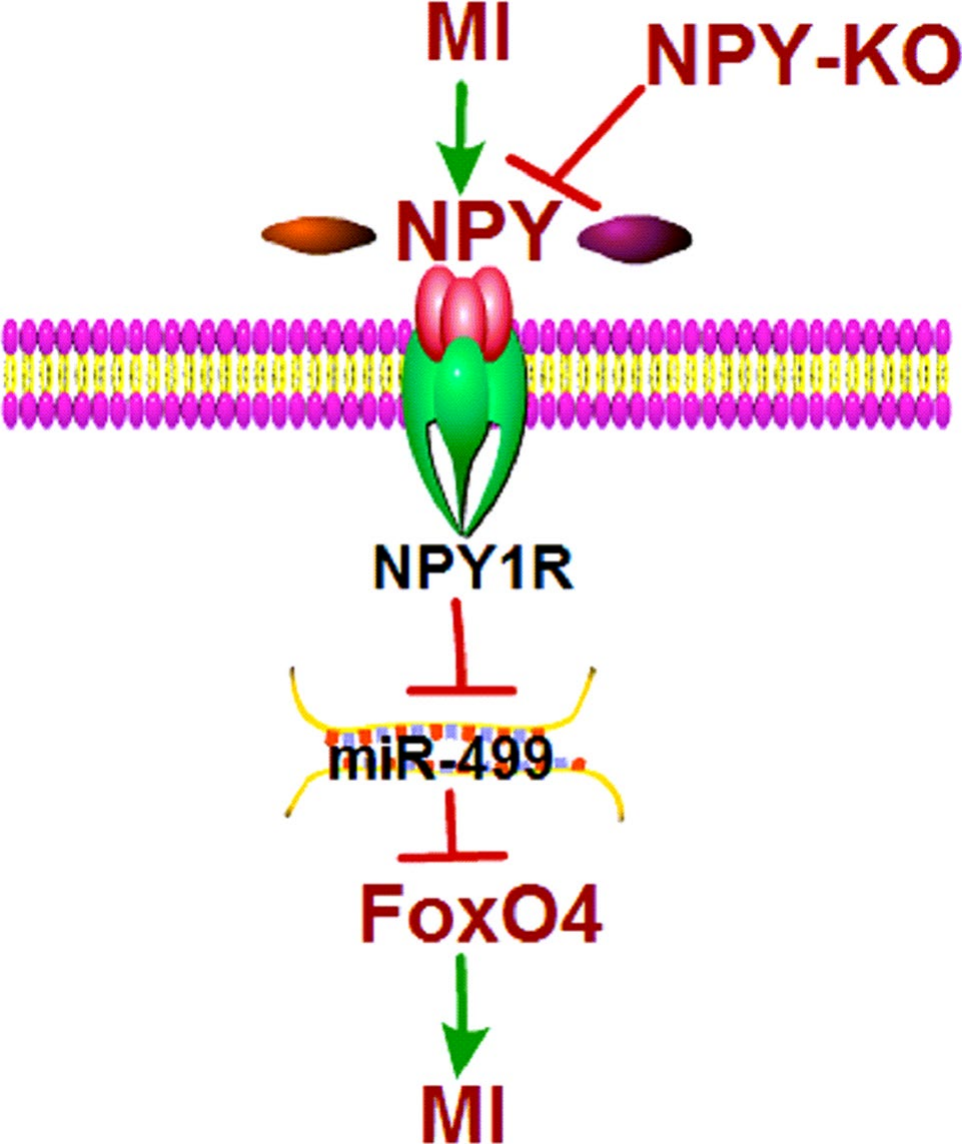
Schematic diagram for the proposed neuropeptide Y mediated myocardial infarction signaling pathways.

The main finding of this study were: (Mozaffarian et al., 2016) NPY was upregulated in both ischemic myocardium and H_2_O_2_-induced cardiomyocytes; (Chiong et al., 2011) NPY-KO reduced infarct size and improved cardiac function in MI rats; (Nabel and Braunwald, 2012) NPY deletion inhibited cardiomyocyte apoptosis in ischemic myocardium and hypoxia/H_2_O_2_-induced cardiomyocytes by NPY1R–miR-499–FoxO4 axis (**Figure 11**). These findings suggest that NPY deletion may be beneficial for repair of ischemic cardiomyocytes.

Previous studies reported that NPY is associated with the progression of various cardiovascular diseases, including hypertension, heart failure, and MI (Dvorakova et al., 2014; Tan et al., 2018). Circulating NPY level is higher in patients with MI and heart failure than the control subjects (Han et al., 1989; Hulting et al., 1990; Ullman et al., 1994). Our findings are in line with these previous results because we found an increase in NPY expression of both ventricular and plasma in MI rats and H_2_O_2_-induced cardiomyocytes. Long-term subcutaneous administration of NPY could induce cardiac dysfunction and cardiac hypertrophy in rats, and NPY treatment could also induce hypertrophy of cardiomyocytes *in vitro* (Chen et al., 2005; Zhang et al., 2015). In addition, NPY impairs cell viability and mitochondrial membrane potential through Ca^2+^ and p38 signaling pathways in neonatal rat cardiomyocytes (Hu et al., 2017). However, the potential role of NPY in MI and MI-induced cardiomyocyte apoptosis remains unclear.

In this study, we evaluated for the first time the role of NPY-KO in cardiac ischemia for 3 days after ligation of left coronary artery induction in rats and 4 h after treatment with H_2_O_2_ and 12 h after treatment with hypoxia in cardiomyocytes. We demonstrated that NPY deletion plays an obvious anti-ischemic injury role *in vivo* or *in vitro* model. Our results showed that myocardial injury caused by ischemia was characterized by increased cardiomyocyte apoptosis, infarct size, and LDH activity and reduced heart function. Hypoxia/H_2_O_2_ reduced cardiomyocyte viability and promoted cell apoptosis. All of ischemia, hypoxia/H_2_O_2_-induced cardiomyocyte injuries were alleviated by NPY deletion, confirming the protective effect of NPY deletion in MI.

MiRNAs are small endogenous nonprotein-coding RNA, which is about 22 nucleotides in length and plays a role in negative regulation of gene expression by pairing with the protein-coding gene mRNAs´ 3´-UTR region (Wang et al., 2016). Recently, it was reported that miRNAs were involved in many pathophysiological processes in the heart, including MI, myocardial hypertrophy, myocardial fibrosis, and heart failure (Condorelli et al., 2014; Wang et al., 2016). Some miRNA expressions were altered in the myocardium of patients with acute MI, including some cardiac-rich and -specific miRNAs including miR-1, miR-133, miR-208, and miR-499 (Chistiakov et al., 2016). Plasma miR-1, miR-208, and miR-499 are potential biomarkers in predicting acute MI in Chinese Han population (Liu et al., 2015). Based on the earlier finding, we found that NPY deletion could reverse the miR-499 expression decrease induced by ischemia/H_2_O_2_. A recent study showed that miR-499 protects cardiomyocytes from H_2_O_2_-induced apoptosis by affecting Pdcd4 and Pacs2 (Wang et al., 2014). FoxO4 is the direct target of miR-499 and promotes cardiac ischemic injuries and myocardial apoptosis (Zhu et al., 2015; Yu et al., 2018). On the basis of these findings, we found that FoxO4 as a direct target of miR-499 and miR-499 mimics significantly inhibited FoxO4 protein expression, and NPY deletion reversed the FoxO4 expression increase induced by H_2_O_2_.

NPY causes MI injury *via* NPY1R, and it is a predominant receptor subtype in the heart (Chottova Dvorakova et al., 2008; Herring et al., 2019). Based on the earlier finding, we found that NPY1R antagonist BIBO3304 could reverse the miR-499 expression decrease and FoxO4 expression increase induced by H_2_O_2_. As expected, BIBO3304 and FoxO4 siRNA could increase cardiomyocyte viability and inhibit cardiomyocyte apoptosis in H9c2 cells, which NPY presented the opposite effect. NPY siRNA inhibited cell apoptosis that were reversed by cotransfection of miR-499 inhibitor in H_2_O_2_-induced cardiomyocyte apoptosis, which demonstrated the negative regulatory effects of NPY on miR-499. Together with the findings in the present study, NPY mediates miR-499–FoxO4 on MI through NPY–NPY1R signaling. It should be noted that NPY–NPY1R–miR-499–FoxO4 signal pathway may merely be one of the mechanisms of NPY. In future work, we will study other potential mechanisms involved in the MI of NPY.

In summary, the present work demonstrated that NPY-KO protected the heart from dysfunction and attenuated ischemic-induced apoptosis by NPY–NPY1R–miR-499–FoxO4 signal pathway in infarct hearts. Our study implies that NPY deletion may be a new treatment option for ischemic heart diseases.

## Data Availability Statement

The datasets for this manuscript are not publicly available because datasets are clearly reported in the manuscript. Requests to access the datasets should be directed to huangwei104@126.com.

## Ethics Statement

All animal agreements were approved by the Animal Care and Use Committee of Harbin Medical University. All experimental procedures are in line with the guidelines for the care and use of experimental animals published by the US NIH (publication, 8th Edition, 2011).

## Author Contributions

WH: funding acquisition, investigation, and writing-original draft preparation. QZ, HQ, PS, CS, and YL: methodology and validation. HS: supervision. HS: project administration and writing-review and editing.

## Funding

This work was supported in part by China Postdoctoral Science Foundation (2017M611401 to WH), Hei longjiang Postdoctoral Science Foundation (LBHZ16244 to WH), the Wu Liande Youth Science Fund of Harbin Medical University (Daqing) (DQWLD201702 to WH), the Fundamental Research Funds for the Provincial Universities of Heilongjiang Province (2017JCZX03 to WH), and the Project of Science and Technology Bureau of Daqing (zdy-2016-075 to WH).

## Conflict of Interest

The authors declare that the research was conducted in the absence of any commercial or financial relationships that could be construed as a potential conflict of interest.
